# Enhanced T-cell activation and chemokine-associated function in CD14-positive cells from venous sinus blood in sub-acute cerebral venous sinus thrombosis

**DOI:** 10.3389/fcell.2024.1488005

**Published:** 2024-11-13

**Authors:** Yu-Zhou Chang, Yu-Qi Song, Hao-Yu Zhu, Jia-Rui Zhang, Xi-Guang Fu, Yi-Long Wang, Ke-Hui Dong, Chu-Han Jiang, Da-Peng Mo, Yu-Peng Zhang

**Affiliations:** ^1^ Department of Neurosurgery, Beijing Neurosurgical Institute, Beijing Tiantan Hospital, Capital Medical University, Beijing, China; ^2^ Department of Neurology, Beijing Tiantan Hospital, Capital Medical University, Beijing, China; ^3^ Interventional Neuroradiology Center, Department of Neurology, Beijing Tiantan Hospital, Capital Medical University, Beijing, China

**Keywords:** cerebral venous sinus thrombosis, idiopathic intracranial hypertension, CD14-positive cell sorting, T cell activation, chemokine-mediated signaling pathway

## Abstract

**Background:**

Patients with sub-acute cerebral venous sinus thrombosis experience (SA.CVST) severe symptoms compared to two other venous sinus-related diseases, including chronic cerebral venous sinus thrombosis (C.CVST) and idiopathic intracranial hypertension (IIH).

**Objective:**

This study aimed to determine whether the different immune reactions in different venous sinuses are related.

**Methods:**

Stagnant blood in the cerebral venous sinuses was extracted by passing a microcatheter and *CD14*-positive cells were sorted by magnetic beads and subjected to RNA-seq sequencing.

**Results:**

Compared to patients with IIH, 128 genes were significantly down-regulated and 373 genes were significantly up-regulated in the sub-acute CVST samples. The functions of these genes were mainly focused on “immune response”, “T cell activation” and “plasma membrane”. Gene Set Enrichment Analysis (GSEA) showed T cell survival and activation-related function significantly unregulated in sub-acute CVST. On the other hand, there were 366 genes down-regulated in chronic CVST and 75 genes up-regulated in chronic CVST. In functional annotation, these differently expressed genes were enriched in the “extracellular region”, “chemokine-mediated signaling pathway” and “immune response”. GSEA analysis confirmed that chemokine-related functions were all up-regulated in sub-acute CVST and monocyte-macrophage adhesion functions were also significantly up-regulated.

**Conclusion:**

This study suggested the *CD14*-positive created an activated immune response in sub-acute CVST.

## 1 Introduction

Cerebral venous sinus thrombosis (CVST) accounts for 0.5%–1% of all strokes and occurs mainly in young female adults (Fan et al., 2020). The clinical prognosis is generally good, but 5%–15% of patients die in the sub-acute phase (Saposnik et al., 2011; Capecchi et al., 2018). The prognosis varies according to the location of thrombosis, the length of the venous sinus involved, the onset of the disease, and the degree of establishment of collateral circulation (Fan et al., 2020). Currently, the first-line treatment is low molecular heparin combined with warfarin sequential therapy. When sub-acute-phase pharmacological treatment fails to alleviate the stenosis of the cerebral venous sinus or the patient’s symptoms, neuro-interventionalists use several strategies such as contact thrombolysis, mechanical thrombectomy, or balloon dilatation to relieve cerebral sinus stenosis of cerebral venous sinus thrombosis. However, 30% of the patients still have no relief of their symptoms (Quealy, 2024; Ilyas et al., 2017). As a result, it is essential to understand more about the characteristics of sub-acute cerebral venous sinus thrombosis, which can help to explore more new therapeutic options.

In thrombosis-related diseases, immune cells are closely involved in the formation and degradation of thrombi (Scherlinger et al., 2023). Local venous stagnation causes hypoxia, activation of vascular endothelial cells, and aggregation of multiple immune cells on the endothelial surface, ultimately leading to the formation of intraventricular thrombi (Tang et al., 2023). Neutrophils can form neutrophil extracellular traps to accelerate thrombus formation, and monocytes and macrophages can secrete cytokines that can be involved in vascular recanalization after venous thrombosis (Subramaniam et al., 2017). However, the specific role of immune cells in cerebral venous sinus thrombosis is not clear. At the same time, the meningeal lymphatics form side by the cerebral venous sinus, which is a bridge between the central nervous system and the peripheral immune system. Thus, the cerebral venous sinus immune environment also affects the immune environment within the brain parenchyma (Louveau et al., 2018). Therefore, it is valuable to clarify the alteration of the immune environment in cerebral venous sinus thrombosis.

*CD14* is the biomarker for monocytes and macrophages (Louveau et al., 2018). *CD14,* working as a biomarker for extracting monocyte-macrophages from blood in liquid biopsies assay, is highly effective in a variety of disease models (Soler et al., 2020; Rudnik et al., 2021; Fuentelsaz-Romero et al., 2021). Liquid biopsies are often less sensitive and less specific because the blood is collected intravenously that far away from the lesion site (Nikanjam et al., 2022). Cerebral venous sinus thrombosis may create a local blood stasis in the venous sinus. Guidewire catheters can reach this area during the intervention, and in this way, locally stagnant blood can be extracted, which carries more immune characteristics of the local lesion than flowing blood.

In this study, we used cerebral venous sinus blood samples from patients with sub-acute cerebral venous sinus thrombosis to compare the transcriptomic profile of *CD14*-positive cells in idiopathic intracranial hypertension and chronic cerebral venous sinus thrombosis to explore the altered immune environment in different disease conditions within the venous sinuses. We found that 1) *CD14*-positive cells in patients with sub-acute cerebral venous sinus thrombosis exhibited stronger immune responses as well as recruitment and activation of T cells compared to IIH and 2) patients with sub-acute cerebral venous sinus thrombosis showed a significant increase in chemokine related functions, cell adhesion and migration comparing to chronic patients within *CD14*-positive cells. This study aimed to clarify the altered immune environment within the cerebral venous sinus under different disease characteristics and to provide a potential therapeutic mechanism for sub-acute cerebral venous sinus thrombosis.

## 2 Materials and methods

### 2.1 Case involvement

This study was approved by the ethical committee of Beijing Tiantan Hospital, Capital Medical University (ethical statement number: KY 2016-039-02). We prospectively enrolled thirty-five patients with cerebral venous sinus-related diseases who came to the Department of Neurointervention of Beijing Tiantan Hospital from December 2021 to June 2022 and underwent interventional procedures (Supplementary Table 1). Ten patients with concomitant infectious diseases were excluded. Five patients were excluded during the extraction process because the number of peripheral blood mononuclear cells (PBMC) was too small. During RNA extraction, two patients were excluded due to low RNA content. A total of 18 patients were finally enrolled, including six patients with sub-acute cerebral venous sinus thrombosis (onset time less than 1 month while more than 1 day), four patients with chronic cerebral venous sinus thrombosis (onset time more than 1 month), and eight patients with idiopathic intracranial hypertension. No acute phase (onset time less than 1 day) patients were involved (Alvis-Miranda et al., 2013).

### 2.2 Blood sampling

After the patient had been given general anesthesia, an intravenous shell was placed in the femoral vein with the assistance of a guide needle, and an 8-F guide catheter was placed through the vein at the level of the jugular bulb. A 6-F intermediate catheter (132-cm Catalyst, Stryker Corporation, Fremont, CA) was then navigated to the cerebral venous sinus lesion via a 260-cm glidewire (Chang et al., 2023). Using a 10 mL syringe, 10 mL of blood was drawn from the lesion section. The blood was pumped into a blood collection tube containing EDTA and placed on ice for transport to the laboratory.

### 2.3 PBMC extraction

Blood from EDTA blood collection tubes was centrifuged, and the 1.5 mL plasma was collected for each patient and stored at −80°C. The lower layer of cells was collected and diluted using DPBS (Thermo, 14040133). The single nucleated cell layer was isolated using Lymphocyte Separation Solution (Corning, 25-072-CV). Erythrocytes were removed from the PBMC using RBC Lysis Buffer (eBioscience, 00-4333-57). PBMCs were washed twice using DPBS. Cells were sorted using a CD14 magnetic bead box (Thermo, 11367D). After centrifugation, *CD14*-positive PBMC was dissolved in Trizol (Thermo, 15596026CN) and stored at −80°C.

### 2.4 RNA extraction and library construction

Total RNA was extracted using Trizol reagent according to the instructions, and RNA purity and quantification were determined using a NanoDrop 2000 spectrophotometer (Thermo Scientific, United States). An Agilent 2,100 Bioanalyzer (Agilent Technologies, Santa Clara, CA, United States) was used to assess RNA integrity. Transcriptome libraries were constructed using the VAHTS Universal V5 RNA-seq Library Prep kit according to the instructions. Transcriptome sequencing and analysis were performed by Shanghai Ouyi Biotechnology Co.

The libraries were sequenced using the Illumina Novaseq 6,000 sequencing platform, and 150 bp double-ended reads were generated. An average of 55,241,667 raw reads were obtained for each sample, and the raw reads in fastq format were processed using the fastp software (Chen et al., 2018). The clean reads were obtained by removing the low-quality reads for subsequent data analysis. HISAT2 (Kim et al., 2015) software was used for reference genome comparison and FPKM (Roberts et al., 2011) calculation and HTSeq-count (Anders et al., 2015) was used to obtain the counts of reads for each gene. PCA analyses of genes (counts) were performed using R (v 3.2.0) as well as mapping to assess the sample biological repeat.

### 2.5 Differentially expressed genes (DEGs) analysis

Differentially expressed genes analysis was performed using DESeq2 (Love et al., 2014) software, where genes that met the thresholds of p-value <0.05 and foldchange >2 or foldchange <0.5 were defined as differentially expressed genes.

Subsequently, gene ontology (GO) (The Gene Ontology Consortium, 2019) and Kyoto Encyclopedia of Genes and Genomes (KEGG) (Kanehisa et al., 2008) Pathway enrichment analyses of differentially expressed genes were performed based on a hypergeometric distribution algorithm for screening significantly enriched functional entries. R (v 3.2.0) was used to plot bar charts, chord charts, or enrichment analysis circle plots for significantly enriched functional entries. Gene set enrichment analysis was performed using GSEA software (Subramanian et al., 2005). Using a predefined set of genes, genes were ranked according to their degree of differential expression in the two sample types and then tested whether the predefined set of genes was enriched at the top or bottom of this ranked list.

### 2.6 RT-PCR analysis

The RNA samples were returned after RNA-seq. There were eight samples remained high quality, including three IIH samples, two SA.CVST samples and 3 C.CVST samples. Three replicates were performed for each sample. 1000ng RNA was converted to cDNA, and SYBR™ Select Master Mix (Thermo) was used to perform quantitative real-time PCR on a 7,500 Fast Real-Time PCR system (Chang et al., 2020). The primer sequences for the genes in this study were listed in Supplementary Table 2.

### 2.7 The enzyme linked immunosorbent assay (ELISA)

The plasma was extracted during PBMC extraction. There were eight plasma samples remained available, including two IIH samples, four SA.CVST samples and 2 C.CVST samples. Three replicates were performed for each sample. CD276 ELISA kit (Proteintech, KE00143) (Huang et al., 2023) and CCL20 ELISA kit (Proteintech, KE00149) (Long et al., 2024) were used to detect the plasma concentration of CD276 and CCL20. Absorbance was detected for each sample at 450 nm.

### 2.8 GSVA score calculation

GSVA is a non-parametric, unsupervised method for assessing the enrichment of transcriptomic gene sets (Hänzelmann et al., 2013). The R package “GSVA” was used to calculate the GSVA score by using neutrophil extracellular traps-related genes. The neutrophil extracellular traps-related genes were collected through the relevant literatures (Luan et al., 2023; Shen et al., 2022) and listed in Supplementary Table 3.

### 2.9 Statistical analysis

One-way ANOVA was used to compare the expression levels of differentially expressed genes among three groups. All statistical analyses were conducted using R v4.4., and Prism 9. A value of p < 0.05 was considered statistically significant.

## 3 Results

### 3.1 Clinical characteristics of patients

**FIGURE 1:**
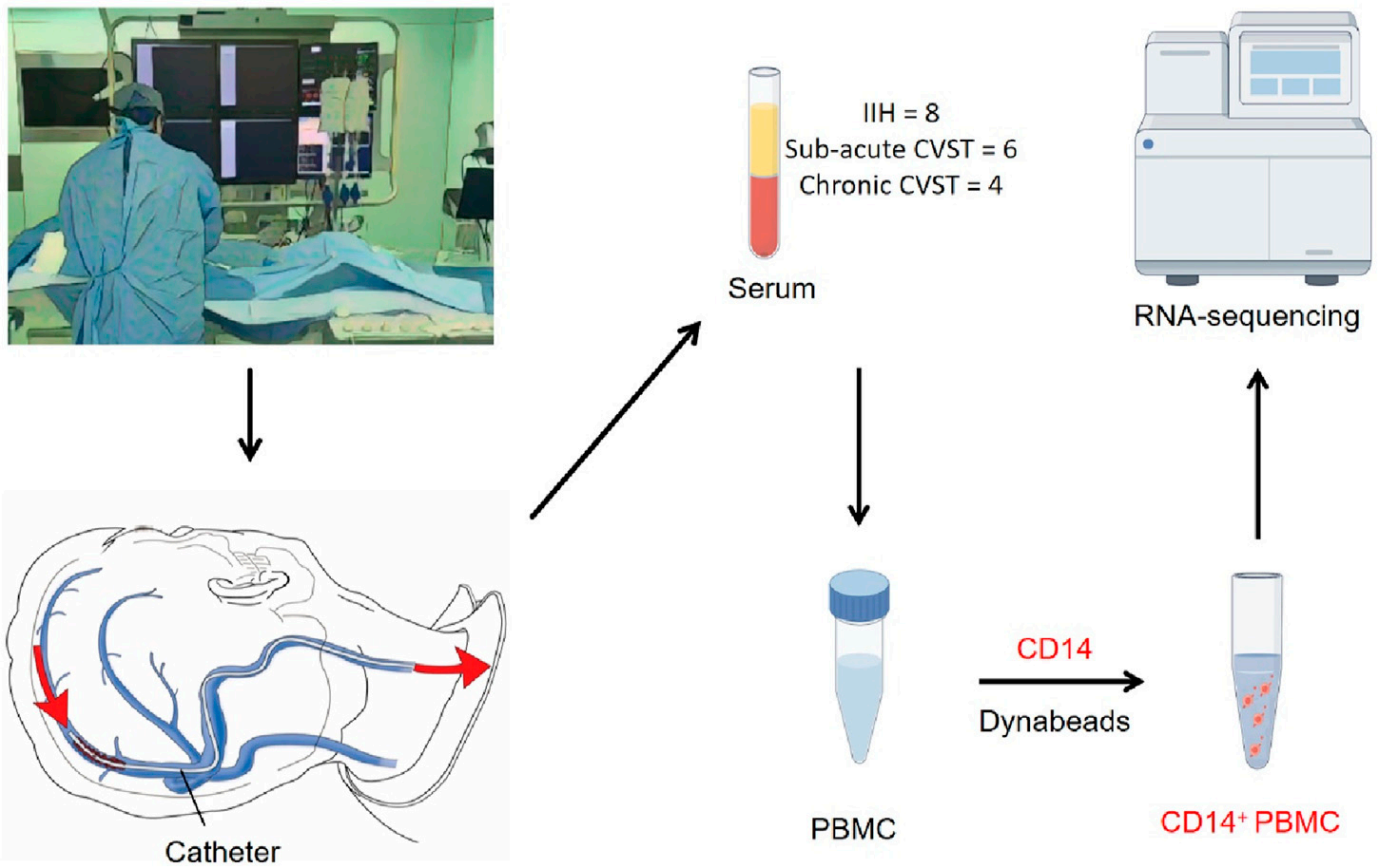
A schematic of the workflow for sample collection. All the samples are extracted during interventional surgery. Catheters were used to collect blood from the venous sinus lesion. PBMC was extracted from blood serum and *CD14*-positive cells were further sorted by *CD14* dynabeads. The sorted *CD14*-positive cells were used in RNA sequencing.

The 18 patients included 6 cases of sub-acute cerebral venous sinus thrombosis (SA.CVST), 4 cases of chronic cerebral venous sinus thrombosis (C.CVST), and 8 cases of idiopathic intracranial hypertension (IIH). Among them, the average age of patients with sub-acute cerebral venous sinus thrombosis was (32.33 ± 5.75), with 50% males (Figure 1). The patients generally had a short onset time, with an average onset time of 7.1 days, accompanied mainly by more serious clinical symptoms. All patients had symptoms of severe headache, five patients were accompanied by severe nausea and vomiting, and two of them had epileptic symptoms. After the intervention surgery, the symptoms were slightly relieved. Four patients with chronic cerebral venous sinus thrombosis, mean age (44.00 ± 20.9), 50% male, all with a disease duration of more than 6 months. The patients suffered a wide range of symptoms, and all patients’ mRS scores decreased below two after the interventional surgery. There were eight patients with idiopathic intracranial hypertension, with the mean age (39.50 ± 13.51), 12.5% male. The duration of the disease was more than 1 month. The most common clinical symptom was blurred vision (six patients), followed by headache (four patients). All of the patient’s symptoms were relieved after receiving interventional treatment. Among the cerebral venous sinus-related diseases, patients with sub-acute cerebral venous sinus thrombosis were more symptomatic, so we further explored the characteristics of their sinus immune environment (Supplementary Table 1).

### 3.2 *CD14*-positive cells in patients with SA.CVST has more robust T-cell recruitment and activation function than IIH

**FIGURE 2:**
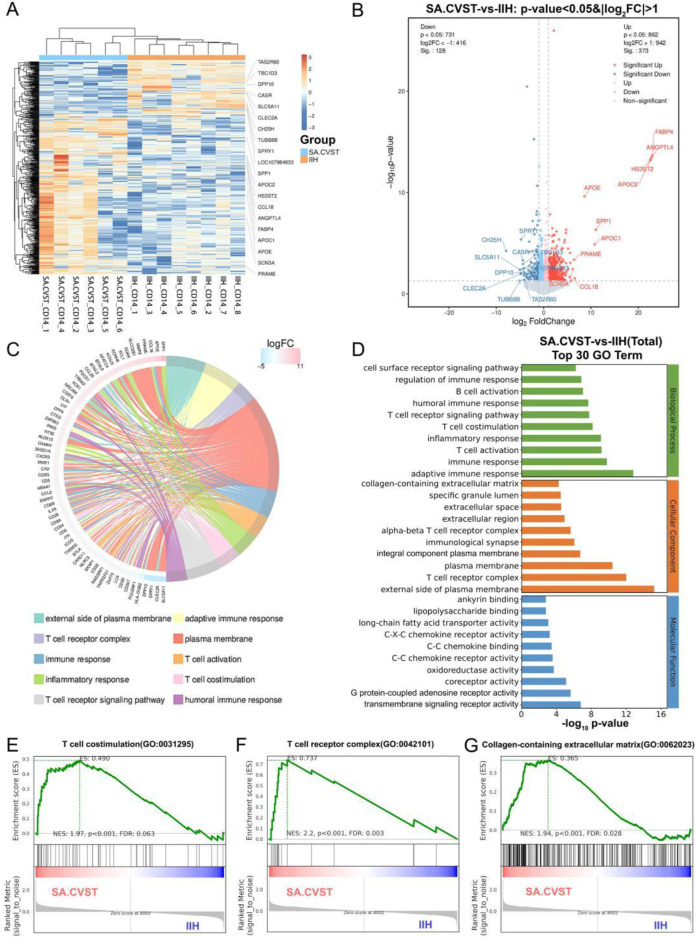
Transcriptomics analysis and GO functional enrichment between sub-acute CVST (SA.CVST) and idiopathic intracranial hypertension (IIH); **(A)** The heatmap of genes expressions of SA.CVST and IIH group; **(B)** The volcano diagram of DEGs of SA.CVST and IIH group; **(C, D)** The GO functional analysis of DEGs; **(E–G)**The GSEA analysis of GO functions in SA.CVST and IIH group.

Patients with sub-acute cerebral venous sinus thrombosis have severe symptoms and poor prognosis. Thus, we first compared *CD14*-positive immune cells in the cerebral venous sinus of patients with sub-acute cerebral venous sinus thrombosis and patients with idiopathic intracranial hypertension. We used a heatmap to portray the transcriptomic data of the patients. We found a significant difference between the two groups, in which genes such as *CLEC2A, TBC1D3*, and *DPP10* were upregulated in the group of idiopathic intracranial hypertension. In contrast, genes such as *SPP1, CCL18* and *HS3ST2* were upregulated in the group of sub-acute cerebral venous sinus thrombosis (Figure 2A).

We used DEseq to calculate the DEGs between the two groups. 373 genes were significantly upregulated and 128 genes were significantly downregulated in patients with sub-acute cerebral venous sinus thrombosis. The significantly upregulated genes included genes closely related to lipid metabolism (*APOE, APOC1*, and *APOC2*), cellular response to cytokine stimulation (*CCL18, XCL1,* and *FABP4*), and P53 downstream signaling pathways (*MMP2, SPP1,* and *GDF15*). Among the significantly downregulated genes were genes associated with positive regulation of GTPase activity (*PREX2, TBC1D3C*, and *TBC1D3*), hydrolase activity (*CASR, SCN2B*, and *DPP10*), and microtubule cytoskeletal organization (*SPRY1, TEKT2,* and *TUBB8B*) (Figure 2B). These different-expression genes resulted in different biological characteristics of *CD14*-positive cells in different diseases.

To fully describe the function of these DEGs above, we performed the gene annotation to these DEGs. We found that most genes were clustered on the “external side of plasma membrane”, “adaptive immune response”, “T cell receptor complex” and “plasma membrane” (Figure 2C). We further analyzed the different GO types and found that the main clustered functions in Biological Processes were immune response, T cell activation, T cell receptor signaling pathway, and cell surface. In the cellular component, the main clusters were plasma membrane and extracellular region-related functions, whereas in the molecular function, the main clusters were chemokine and cytokine receptor functions and fatty acid metabolism (Figure 2D). To further verify whether these functions are activated or inhibited in cerebral venous sinus thrombosis, we performed GSEA analysis and found that “T cell costimulation”, “Collagen-containing extracellular matrix” and “Nuclear hormone receptor binding” were all significantly elevated in the sub-acute cerebral venous sinus thrombosis group (Figures 2E–G).

In this section, SA.CVST was used to compare with IIH. Although these two diseases had the common feature that all lead to stagnation of blood flow in the venous sinuses, *CD14*-positive monocyte-macrophages showed different activation characteristics. In patients with A.CVST, *CD14*-positive cells upregulate genes and signaling pathways associated with innate immune activation, as well as activating T cells to create an immune-activated microenvironment. In contrast, in IIH, the activated genes are mainly focused on maintaining cellular homeostasis.

### 3.3 *CD14*-positive cells in SA.CVST patients have more substantial chemokine-related functions than those with C. CVST

**FIGURE 3:**
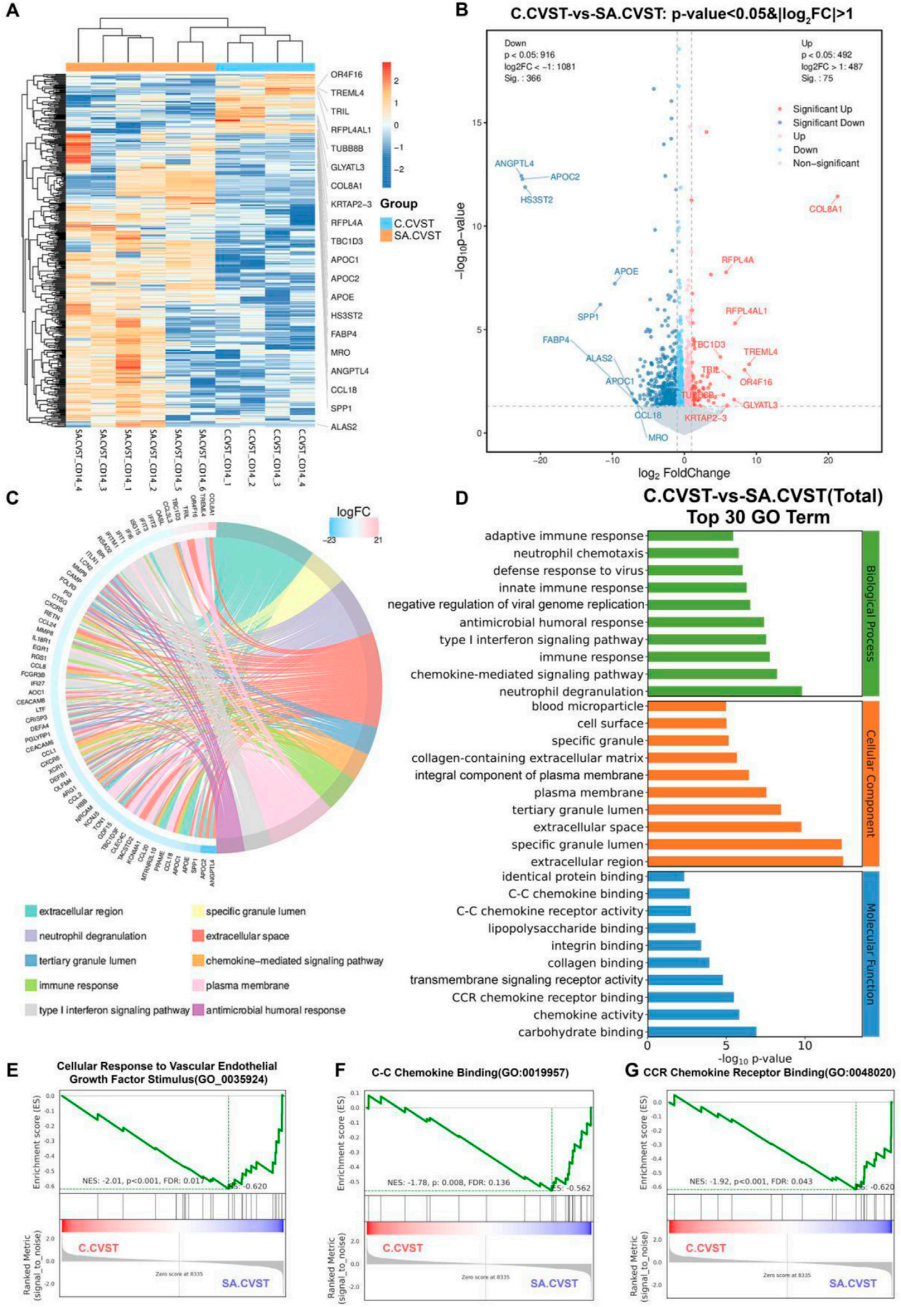
Transcriptomics analysis and GO functional enrichment between sub-acute SA.CVST and chronic CVST (C.CVST); **(A)** The heatmap of gene expressions of SA.CVST and C.CVST; **(B)** The volcano diagram of DEGs of SA.CVST and C.CVST group; **(C, D)** The GO functional analysis of DEGs; **(E–G)** The GSEA analysis of GO functions in SA.CVST and C.CVST group.

To further clarify the alterations in the different phases of cerebral venous sinus thrombosis microenvironment, we compared *CD14*-positive cells in patients with sub-acute cerebral venous sinus thrombosis with those in patients with chronic cerebral venous sinus thrombosis of more than 6 months duration. We portrayed the expression profiles of *CD14*-positive cells in patients with sub-acute and chronic cerebral venous sinus thrombosis by heatmap. There was a clear distinction between the two expression profiles, in which the expression of *APOC2, ANGPTL4*, and *APOE* was elevated in the sub-acute cerebral venous sinus thrombosis group, whereas the expression of the genes *OR4F16, TREML4,* and *TRIL* was elevated in the chronic cerebral venous sinus thrombosis group (Figure 3A).

We further analyzed the DEGs between the two groups, showing that 75 genes were significantly upregulated in patients with chronic cerebral venous sinus thrombosis, whereas 366 genes were significantly down-regulated. Among the significantly upregulated genes were genes related to positive regulation of cellular catabolic process-related genes (*PNLDC1, REPL4A, REPL4AL1*) and calcium signaling pathway-related genes (*ADCY2*, OXTR, *RET*). On the other hand, significantly downregulated genes included regulation of vesicle-mediated transport-related genes (*APOC1, APOC2, APOE*), cytokine-cytokine receptor interaction-related genes (*CCL18, CCL20, GDF15*), and response to hypoxia-related genes (*ALAS2, KCNMA1, ANGPTL4*) (Figure 3B).

We functionally clustered the DEGs and the clusters were most functionally enriched in “extracellular region,” “specific granule lumen,” “neutrophil degranulation,” and “extracellular space” (Figure 3C). Among the biological processes, the most enriched terms related to various immune responses and neutrophil-related functions. The most relevant functions for cellular components included the cell surface, extracellular spaces and regions, and the plasma membrane. Considering molecular functions, C-C chemokine binding, CCR chemokine receptor, and collagen binding were highly enriched (Figure 3D). In GSEA analysis, ‘cellular response to vascular endothelial growth factor stimulation’, ‘C-C chemokine’, and ‘CCR chemokine receptor binding’ functions were upregulated in SA.CVST patients. These annotations suggested that *CD14*-positive cells were more sensitive to chemokine activation in SA.CVST patients (Figures 3E, F). C-C Chemokine and CCR Chemokine Receptor Bindings are critical for vascular endothelial interactions with monocytes and macrophages, which can help monocyte-macrophages escape the vasculature into the brain tissue. Thus, *CD14*-positive cells are more sensitive to chemokine stimulation in patients with sub-acute cerebral venous sinus thrombosis.

### 3.4 Validation of DEGs expression and T cell activation-related function

**FIGURE 4:**
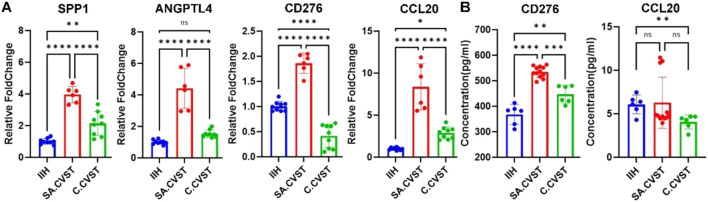
Validating some important DEGs expressions and T cell activation-related functions. **(A)** The expression of *SPP1, ANGPTL4, CD276* and *CCL20* in *CD14*-positive cells of IIH, SA.CVST and C.CVST samples. **(B)** The concentration of *CD276* and *CCL20* in the plasma of IIH, SA.CVST and C.CVST patients.

To further support our findings in SA.CVST group, the RNA-seq return RNA samples were used to validate the top different expressed genes. There were eight RNA samples that remained high quality. Metabolism-related genes (*SPP1, ANGPTL4*) and immunomodulatory molecule-related genes *(CD276, CCL20)* were detected by RT-PCR. Consistent with RNA-seq data, *SPP1, ANGPTL4, CD276* and *CCL20* were upregulated in SA.CVST groups compared to IIH and C.CVST groups (Figure 4A).

T cell activation functions were validated by ELISA assay. Eight patients’ plasma was used to detect T-cell activation-related chemokines. According to our RNA-seq results, *CD276* and *CCL20* were chose as the related chemokines. In the *CD276* ELISA assay, the concentration of *CD276* was significantly increased in SA.CVST patients’ plasma compared to IIH and C.CVST. In C.CVST patients’ plasma, the concentration of *CD276* also increased compared to IIH. Regarding the *CCL20* ELISA assay, the concentration of *CCL20* showed no significant difference between SA.CVST and the other two groups. In C.CVST patients, the plasma *CCL20* concentration decreased compared to IIH group. The concentrations of *CCL20* remained low while the variation of different samples was severe. *CD276* were 100-fold higher compared to those of *CCL20*. In all, the concentrations of *CD276* were higher in SA.CVST patients’ plasma, which might help T cell activation in SA.CVST groups (Figure 4B).

### 3.5 SA.CVST CD14-positive cells express higher neutrophil extracellular traps-related genes

**FIGURE 5:**
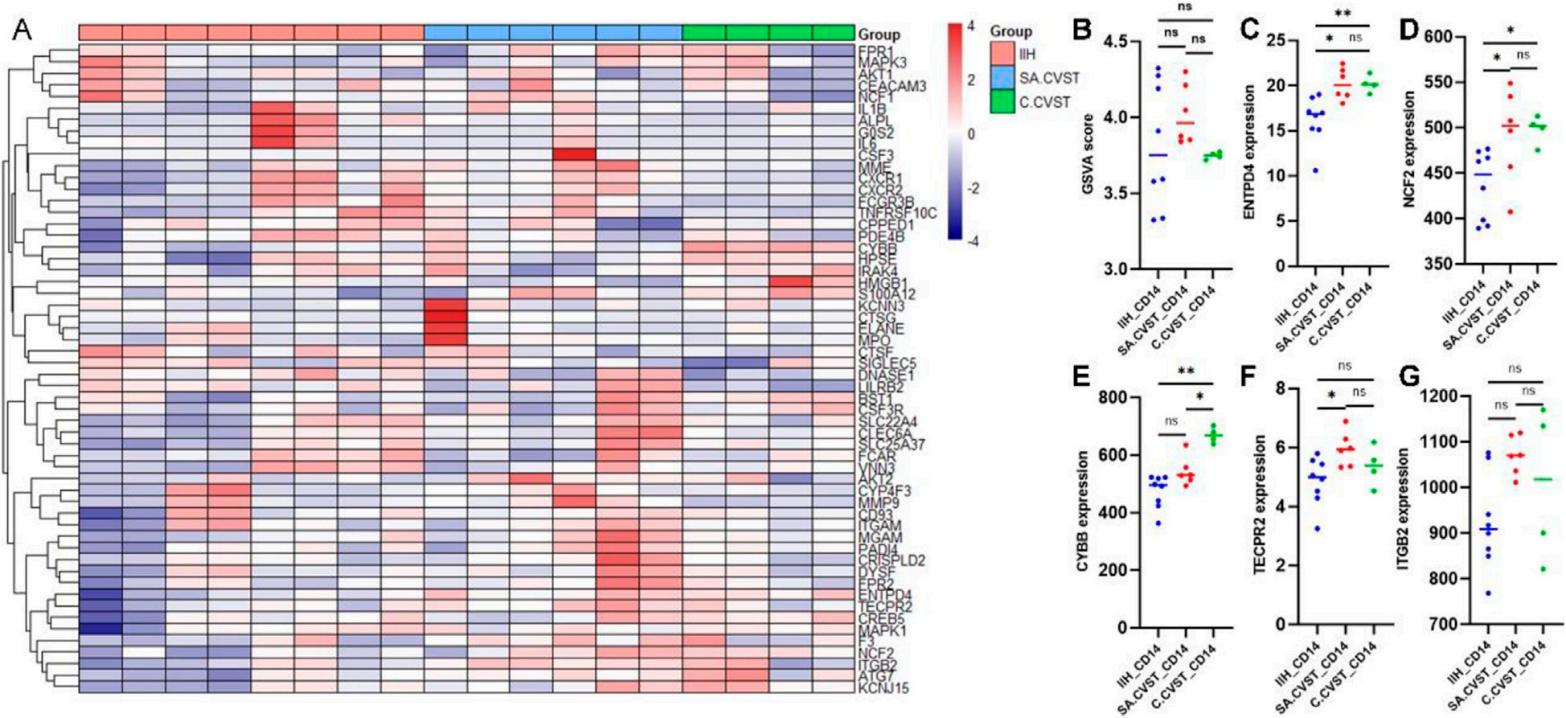
Neutrophil extracellular traps-related gene expression among IIH, SA.CVST and C.CVST. **(A)** The heatmap of neutrophil extracellular traps-related genes among different groups; **(B)** The GSVA score of neutrophil extracellular traps among different groups; **(C–G)** *ENTPD4* **(C)** *, NCF2* **(D)** *, CYBB* **(E)** *, TECPR2* **(F)** *, and ITGB2* **(G)** gene expression among different groups.

As mentioned in previous studies, neutrophil extracellular traps (NETS) played an important role in mediating CVST formation (Jin et al., 2022). CD14-postive monocyte-macrophage also participated in neutrophil extracellular traps formation. Thus, neutrophil extracellular traps-related genes are examined in our RNA sequencing data. As shown in the heatmap, the NETS-related genes are not specifically distributed in SA.CVST or C.CVST groups (Figure 5A). NETS GSVA scores were compared in all the samples and no significant difference was found between the different groups (Figure 5B). Several most important NETS-related genes (*ENTPD4, NCF2, CYBB, TECPR2, ITGB2*) were examined individually (Figures 5C–G). *ENTPD4* and *NCF2* up-regulated in SA.CVST and C.CVST groups compared to IIH. *CYBB* up-regulated in C.CVST groups compared to SA.CVST and IIH groups. *TECPR2* upregulated in SA.CVST group comparing IIH group. *ITGB2* expression has no difference between the three groups.

## 4 Discussion

Cerebral venous sinus thrombosis (CVST) is a cerebrovascular disease that involves the venous sinus (Fan et al., 2020). Due to various causes of venous sinus thrombosis, venous outflow channels are blocked, which results in cerebral venous sinus hypertension, cerebral venous drainage, and cerebrospinal fluid absorption impairment (Capecchi et al., 2018). As a result, the intracranial pressure increases significantly which leads to cerebral edema, cerebral hemorrhage, or venous infarction (de Bruijn et al., 2001). Patients with these complications can be seriously life-threatening. In contrast, patients with chronic cerebral venous sinus thrombosis have light symptoms and rarely die. In our study, all SA.CVST patients suffered severe headaches and four of six patients witnessed severe nausea and projectile vomiting. The rate of epilepsy onset also increased in SA.CVST group, which also supports SA.CVST patients developed neuronal damage, a more aggravated symptom.

In this study, to better portray the altered immune environment within A.CVST, we used another non-thrombotic disease involving cerebral venous sinuses, idiopathic intracranial hypertension, as a control group. IIH is primarily due to cerebral venous sinus vascular degeneration or underdevelopment, which produces stagnation or turbulence in the venous sinuses (Zhang et al., 2023). This is an anatomically structured disease and does not significantly affect the immune environment within the venous sinuses. IIH tends to cause persistent blurred vision or severe headaches but rarely results in death. The primary treatment of IIH relies on the direct opening of the venous sinus vessels by interventional means using balloons and stents creating a path for neuro-interventionalists to collect the local blood samples in the venous sinus (Zhang et al., 2022).

Liquid biopsies of blood are widely used in the diagnosing, treating, and monitoring of a wide range of diseases due to their convenient sample acquisition. In brain tumors, liquid biopsy detects circulating tumor cells in blood samples and monitors of tumor progression or recurrence by cross-checking the number of tumor cells in the blood with imaging features (Alix-Panabières and Pantel, 2021). In studying other intracranial diseases, monitoring Tau protein and amyloid-β exosomes within the blood can provide early diagnosis of Alzheimer’s disease. Based on proteomic data in the plasma, the immune microenvironment can be analyzed to speculate on the likelihood that the patient has Alzheimer’s disease (Jia et al., 2019; Wang et al., 2023). However, liquid biopsy is less sensitive and specific because it is taken from peripheral blood, which is far from the lesion site. In this study, we collected blood from the lesion site through an interventional catheter during the interventional procedure. The PBMC of the local blood carries more lesion-related transcriptomic alterations, thus increasing the sensitivity and specificity of the liquid biopsy. We extracted the cells in the PBMC of the patient’s lesion site and analyzed the different functions presented by the cells in different disease states. The transcriptome of the cells was sequenced by RNA-seq to tap the altered gene expression of the PBMC in different diseases, which found factors influencing the response to drug therapy for sub-acute cerebral venous sinus thrombosis.

The cerebral venous sinus is constructed in the dura mater and is an important essential for cranial blood reflux. Meningeal lymphatics along the venous sinus is a vital communication region for immune cells of the central immune system and the peripheral blood system, which closely monitors the changes in the cerebral immune environment in physiological and pathological states (Da Mesquita et al., 2018; Rustenhoven et al., 2021; Louveau et al., 2015). A study related to subarachnoid hemorrhage showed that blood clots can block the meningeal lymphatics, affecting meningeal lymphatics drainage function, allowing the undrained cerebrospinal fluid to enter the perivascular space, damaging aquaporin 4, leading to excessive accumulation of toxic and inflammatory metabolites. In turn, this phenomenon may induce long-term brain damage (Liu et al., 2020). Besides, monocyte-macrophages and T-lymphocytes, involved in immune surveillance and elimination, are mainly derived from dural sinusoids and blood vessels. Therefore, the dural sinuses and vessels affect the function of meningeal lymphatics (Papadopoulos et al., 2020). To further explore the immune alterations in the cerebral venous sinus in disease conditions, we extracted *CD14*-positive cells from PBMC by magnetic beads. *CD14* is a surface marker for monocyte-macrophages, which is closely related to cerebral venous sinus function and thrombosis and is more suitable for reflecting the immune environment of cerebral venous sinus thrombosis.

By comparing sub-acute cerebral venous sinus thrombosis with idiopathic cranial hypertension, we found that the upregulated genes of *CD14*-positive cells in sub-acute cerebral venous sinus thrombosis mainly contained signaling pathways closely related to immune activation, including lipid metabolism, cytokine activation, and transcriptional regulation while the downregulated genes were related to metabolic enzymes such as GTPase and hydrolase. As found in Guo-Chang Hu’s research, inactivation of GTPase in macrophages was found to promote phagocytosis of neutrophils (Jiang et al., 2017). After sub-acute cerebral venous sinus thrombosis, GTPase activity was inhibited in activated macrophages, accelerating macrophage phagocytosis of neutrophils and participating in thrombus degradation. We performed functional clustering and GSEA analysis of differential genes and found that the most critical functional enrichment of *CD14*-positive cells activated by sub-acute cerebral venous sinus thrombosis was T cell recruitment and activation. Traditionally, thrombosis has been understood primarily in terms of platelet aggregation and coagulation factors, but recent studies have highlighted the important involvement of T cells in this process (Zahran et al., 2024). T cells experience altered metabolism levels during thrombosis, with increased cholesterol metabolism and decreased glycolysis. Based on the metabolic alterations, the functional phenotypes of the T cells are also altered at a molecular level. The proportion of effector T (Teff) cells increased significantly after thrombosis. They were also stimulated by innate immune cytokines to transform from vascular barrier-protective cells to vascular-injury cells secreting IL-17A, IL-22, and IFN-g (Burkett et al., 2015). Thus, in sub-acute cerebral venous sinus thrombosis, activated *CD14*-positive cells stimulate T-cell activation, further exacerbating vascular endothelial cell injury consistently. This evidence described one possible reason for severe symptoms in sub-acute cerebral venous sinus thrombosis patients.

We further compared the differences in the expression of the transcriptome of *CD14*-positive cells in sub-acute and chronic cerebral venous sinus thrombosis, and we found that in sub-acute cerebral venous sinus thrombotic cells, cytokine-mediated genes for vesicle-dependent cytosolic cytotoxicity, which is closely related to monocyte-macrophage function, were significantly upregulated, as well as the hypoxia-associated gene expression; whereas significantly down-regulated genes were associated with intracellular catabolism and calcium channels. We performed functional enrichment of cellular functions and found that cytokine and chemokine functions were closely up-regulated. Monocyte-macrophages accumulate to lesion sites via chemokines, while different chemokines cause monocyte-macrophages to differentiate into different phenotypes (Bai et al., 2023). Meanwhile, the CCR family can mediate monocyte-macrophage interactions with endothelial cells, which is a mechanism for how to migrate out of the vessels into the brain parenchyma (Sun et al., 2021). In several studies, *CCR2-CCL2* can help the monocyte-macrophages evade vascular endothelial cells into parenchyma to produce ischemia-reperfusion injury (Geng et al., 2022) or post-epilepsy neuronal injury (Varvel et al., 2016). In this study, monocyte-macrophages expressed higher chemokines in the sub-acute phase. Therefore, more monocyte-macrophages were more likely to enter the brain parenchyma by chemokine recruitment, which may account for the severity of symptoms in the sub-acute phase.

There are some limitations in this study. Firstly, this study’s sample size is relatively small because it is difficult to collect samples from venous sinus through catheters. As a result, we can only describe the expression changes of *CD14*-positive cells in the cerebral venous sinus in different disease states, while we cannot analyze the effects of these changes on the prognosis of the patients. Besides, the tissue samples of the cerebral parenchyma and the meningeal lymphatic vessels were not available in this study, and we could not determine the effects of the expression changes of immune cells in the cerebral venous sinus on the immune environment in the cerebral parenchyma and meningeal lymphatic vessels. Based on the present results, we will further explore the effects of cerebral venous sinus-related diseases on the brain parenchyma and meningeal lymphatics in animal models.

## 5 Conclusion

In this study, by comparing sub-acute and chronic cerebral venous sinus thrombosis and idiopathic intracranial hypertension *CD14*-positive cells, we found that sub-acute cerebral venous sinus thrombosis monocyte-macrophages expressed a more vital immune activation state, played an essential role in recruiting and stimulating T-cell functions, and expressed higher chemokines and cytokines with significantly enhanced cell migration functions. These above results depicted changes in the immune environment within the sinus of sub-acute cerebral venous sinus thrombosis and provided a basis for further exploration of potential therapeutic modalities.

## Data Availability

The data reported in this paper have been deposited in the OMIX, China National Center for Bioinformation/Beijing Institute of Genomics, Chinese Academy of Sciences ( https://ngdc.cncb.ac.cn/omix/ with accession OMIX007793).
